# Development, validation and translation of cardiopulmonary resuscitation and automated external defibrillator training and placement bilingual questionnaire

**DOI:** 10.1186/s13104-019-4698-x

**Published:** 2019-10-21

**Authors:** Keng Sheng Chew, Siew Yee Liaw, Ahmad Zulkarnain Ahmad Zahedi, Shirly Siew Ling Wong, Nariman Singmamae, Dev Nath Kaushal, Hiang Chuan Chan

**Affiliations:** 1 Faculty of Medicine and Health Sciences, Universiti Malaysia Sarawak, Kota Samarahan, Malaysia; 2 Emergency Medicine and Trauma Department, Sarawak General Hospital, Kuching, Malaysia; 3 Faculty of Medicine, Universiti of Malaya, Kuala Lumpur, Malaysia; 4 Faculty of Economics & Business, Universiti Malaysia Sarawak, Kota Samarahan, Malaysia

**Keywords:** Construct validation, Exploratory factor analysis, Confirmatory factor analysis, Cardiopulmonary resuscitation, Automated external defibrillator, Forward translation, Backward translation

## Abstract

**Objectives:**

This paper describes the development and translation of a questionnaire purported to measure (1) the perception of the placement strategy of automated external defibrillator, (2) the perception on the importance of bystander cardiopulmonary resuscitation and automated external defibrillator (3) the perception on the confidence and willingness to apply these two lifesaving interventions as well as (4) the fears and concerns in applying these two interventions. For construct validation, exploratory factor analysis was performed using principal axis factoring and promax oblique rotation and confirmatory factor analysis performed using partial least square.

**Results:**

Five factors with eigenvalue > 1 were identified. Pattern matrix analysis showed that all items were loaded into the factors with factor loading > 0.4. One item was subsequently removed as Cronbach’s alpha > 0.9 which indicates redundancy. Confirmatory factor analysis demonstrated acceptable factor loadings except for one item which was subsequently removed. Internal consistency and discriminant validity was deemed acceptable with no significant cross-loading.

## Introduction

While a number of questionnaire measuring the various dimensions of bystander cardiopulmonary resuscitation (CPR) [1–4] and AED [2–7] have been published, there are few questionnaires that combine both bystander CPR and automated external defibrillator (AED) measurement [8, 9]. We reported the development and construct validation (exploratory factor analysis, EFA and confirmatory factor analysis, CFA) of a bilingual (English and local Malay language) questionnaire that comprehensively measures four objectives: (1) the perception of AED placement strategy, (2) the perception on the importance of bystander CPR and AED, (3) the perception on the confidence and willingness to apply these two lifesaving interventions as well as (4) the fears and concerns in applying these two interventions.

## Main text

### Methods

#### Participants

For EFA, 184 administrative employees from Universiti Malaysia Sarawak (UNIMAS) (who were participants of CPR and AED workshops), responded to the questionnaire. Healthcare employees such as doctors, nurses and paramedic staff were excluded from this study. The mean age of the participants was 37.6 years (standard deviations ± 6.85); and 100 of them (54.3%) were male participants. The number of participants who responded exceeded the estimated sample size according to Costello and Osborne [10] (i.e., minimum of 5 responses per item). As there were 22 items to be validated, the estimated sample size was 110 participants. For CFA, 100 medical doctors from the emergency department of Sarawak General Hospital (age range from 28 years to 32 years old) and 122 final year medical students from UNIMAS (age range from 23 to 25 years old) and who had been trained in CPR and AED, responded to the questionnaire. Convenient sampling was applied in recruiting the participants.

### Materials

The EFA was performed in Statistical Package for the Social Sciences (SPSS) software using principal axis factoring as the extraction method. For CFA, reflective measurement modelling using partial least square was performed using SMART-PLS software. For translation of the questionnaire from English language into the local Malay language, the International Society for Pharmacoeconomics and Outcomes Research (ISPOR) principles of good translation practice for cultural adaption was adopted as the working framework [11]. According to the ISPOR principles, a good translation has the following steps: (1) forward translation, (2) reconciliation (3) backward translation (4) harmonization [11]. In the forward translation stage, two authors who are fluent in both English and Malay language (SYL, DNK) independently translated the questionnaire into Malay language. After completion of the initial Malay language translation, these 2 authors of this paper then discussed and resolved any discrepancy of the translations (reconciliation). Subsequently, an independent language expert who is fluent in both languages were invited to translate the Malay language version back to English language (backward translation). Finally the original English version and the backward English version would be compared to check for significant discrepancy (“harmonization”).

### Procedures

Ethical approval was obtained from the Medical Research and Ethics Committee, Ministry Of Health Malaysia and the study was registered under the Malaysian National Medical Research Register (NMRR, website URL: www.nmrr.gov.my) with the research number of NMRR-16-696-39041. Written informed consent was obtained from all participants prior to their participation in this study.

With regards to the development and construct validation of the questionnaire, a preliminary version of the checklist was first constructed based on previous works [1, 3–9, 12] as well as opinions from four authors of this paper (KSC, SYL, NS and DNK). A modified Delphi method via iterative email communications and face-to-face discussion sessions were conducted. The purpose of these discussions was to come up with a list of the pertinent items purported to measure (1) the perception of the importance of bystander CPR and public access AED, (2) the perception of the confidence and willingness of bystanders to apply these two lifesaving interventions as well as (3) fears and concerns of bystander that may deter their willingness and (4) the perception of the placement strategies of these AED.

Once the preliminary list of items was determined, the participants were asked to rank the items in a Likert scale of four, ranging from “1 = strongly disagree” to “4 = strongly agree”. An initial run of EFA was performed in order to determine the number of factors to be fixed (eigenvalue > 1). After fixing the number of factors, re-run of EFA was then performed to determine the factor loadings of the items as well as to identify items that may need to be removed. Promax oblique rotation was again used. In the pattern matrix, factor loading with cut-off value of < 0.4 was used as the criteria to determine whether an item was to be removed or not [13]. The communality value, which indicates convergent validity of the items, was set at 0.25. Finally, the Cronbach’s alpha coefficients (with > 0.6 cut-off value) were then checked to evaluate the degree of internal consistency of the items in each construct or factor [13].

With regards to CFA, convergent validity, internal consistency, and discriminant validity were determined using partial least square (PLS). For internal consistency, the composite reliability was determined, whereas for convergent validity, factor loadings and average variance extracted (AVE) were determined [14]. In this regard, factor loading of > 0.70 is used as the cut-off point; whereas for item with factor loading between 0.4 and 0.7, the effect of its removal on the overall AVE would be considered. If the removal of the item improves the AVE of the factor, the item would be removed unless the item is a priori determined by the authors to be of critical importance in terms of content validity [14, 15].

With regards to the translation process, two of the authors of this paper (SYL, DNK) independently translated the original English version into the targeted Malay language (“forward translation”) version. These two authors are proficient in both English language and Malay language. SYL is a medical doctor working in the emergency department of Sarawak General Hospital while DNK is a nursing educator from UNIMAS. These 2 authors then compared their versions of the translation with the aim of merging their versions into a single forward translation version (“reconciliation” stage). The backward translation was performed by an independent translator who is proficient in both English and Malay languages and who has vast experience in doing journalistic translation work in two languages. After completion of the backward translation, three authors (SYL, DNK, NS and KSC) then compared the back translated English version with the original English version to check for significant discrepancy (“harmonization”). Any significant discrepancy would be discussed, and revised if deemed necessary. In the unlikely event where there is discrepancy in which the authors could not amicably resolve, an independent language expert who is fluent in both English and Malay languages would be called in.

### Results

**Fig. 1:**
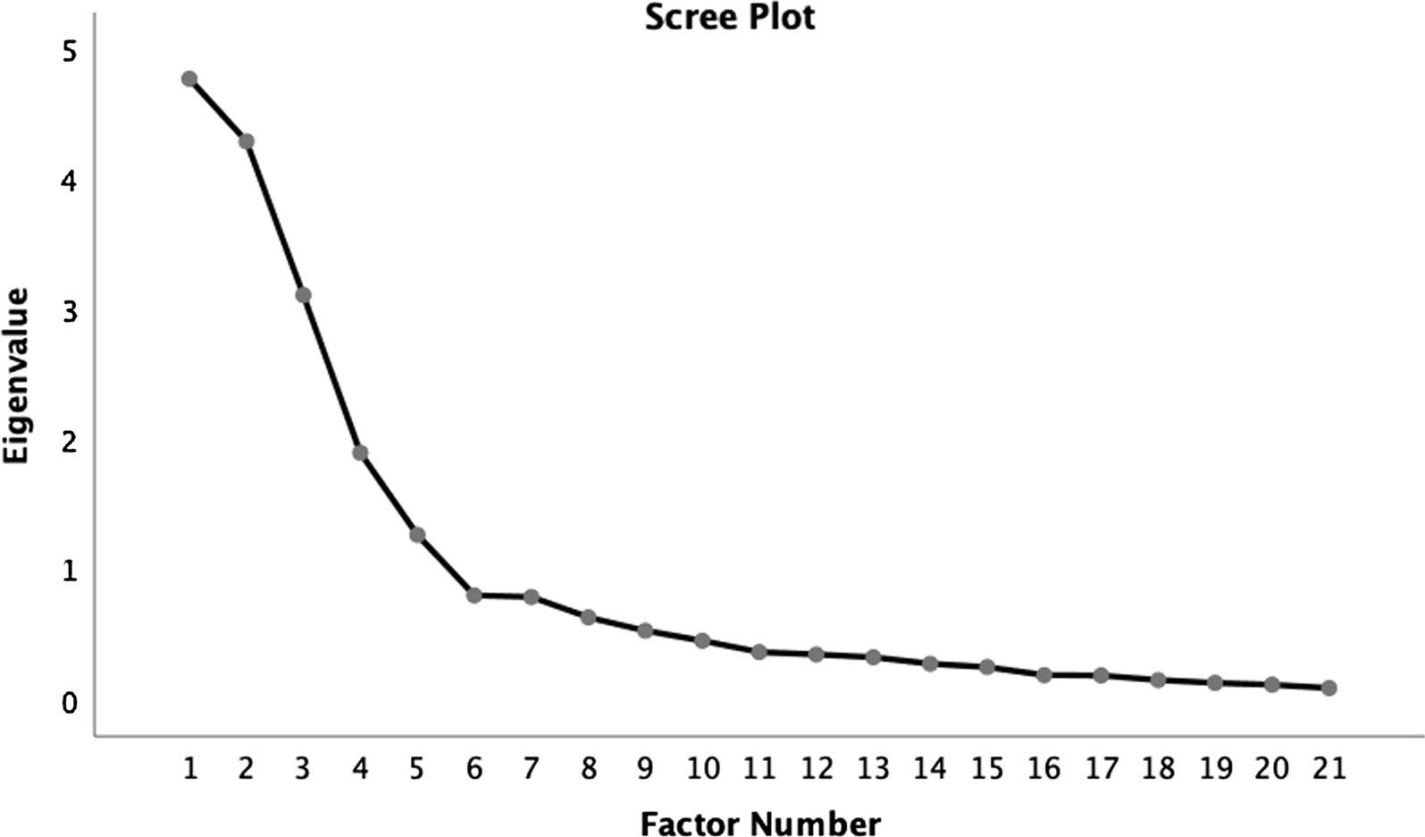
Screen plot shows 5 factors with Eigenvalue > 1

With regards to the development and construct validation of the questionnaire, the Kaiser–Meyer–Olkin measure of sampling adequacy was 0.79 indicating sampling adequacy for EFA. The p-value for Bartlett’s test of sphericity was < 0.001 indicating that there are worthwhile correlations among the items based on the correlation matrix. There are five factors with initial Eigenvalue > 1 (also demonstrated in scree plot, see Fig. 1). The re-run of EFA using promax oblique rotation showed that the communalities of all items > 0.25. Pattern matrix analysis showed that all items were loaded into the factors with factor loading > 0.4 (see Table 1 for the detailed factor loadings after the initial run of EFA). The Cronbach’s alpha value for Factor 1 was 0.955 with 6 items loaded into it, suggesting that there are redundant items. When checked for redundancy, it was noted that item “The directions that point to the location of the AED are clear” carries very similar meaning with item “The signage that shows the location of the AED is clear”. Hence, item “The directions that point to the location of the AED are clear” was removed. After removal, the internal consistency of Factor 1 is still good with Cronbach’s alpha of 0.942. The internal consistency for Factor 2, Factor 3, Factor 4 and Factor 5 were also good with Cronbach’s alpha of 0.855, 0.787, 0.914 and 0.893 respectively. The corrected item-total correlation of all items ranged from 0.41 to 0.89. No negative correlation was noted. A re-run of the EFA after deletion of item “The directions that point to the location of the AED are clear” was subsequently performed and showed that the factor loadings are still good.

**Table 1:** The factor loadings with pattern coefficient values after the initial run of exploratory factor analysis

Item	Factor				
	1	2	3	4	5
The AED is clearly visible	0.911				
The directions that point to the location of the AED are clear^a^	0.896				
The signage that shows the location of the AED is clear	0.936				
The AED is located in a location that is easily accessible at all times (including after office hours)	0.896				
The steps in the AED instructional poster on how to use the AED are easy to follow	0.848				
The AED is located at a secure site	0.819				
CPR and AED are important in saving life		0.724			
It is important for an AED to be available in the place where I work		0.880			
Using an AED is important on any unresponsive victims		0.774			
Person who handles an AED requires formal training		0.691			
AED practice drills should be performed on a regular basis		0.609			
I am concerned in getting infection from the victim when performing CPR				0.500	
I am concerned in injuring the victim when performing CPR				0.499	
I am concerned in injuring myself when performing CPR				0.801	
I am concerned in injuring the victim if I use an AED device during CPR				0.525	
I am concerned in injuring myself if I use an AED device during CPR				0.770	
I am concerned that I might be sued if I perform emergency CPR inappropriately					0.909
I am concerned that I might be sued if I used an AED inappropriately					0.948
I am confident to perform CPR			0.842		
I am confident to use an AED			0.884		
I am confident in recognizing victim with no signs of life			0.743		
I will not hesitate to do CPR and to use an AED on an unresponsive victim			0.849		

Extraction method: principal axis factoring. rotation method: Promax with Kaiser normalization

^a^This item was removed in the final version of the questionnaire

Based on the items that are loaded into the 5 factors, Factor 1 is labelled as “perception of AED placement strategies”, Factor 2 as “perception of importance of CPR and AED”, Factor 3 as “concerns of injuring victims during CPR and AED”, Factor 4 as “concerns of legality in performing CPR and AED” and Factor 5 as “confidence and willingness to perform CPR and AED”.

With regards to the CFA, the factor loadings of all items ranged between 0.48 and 0.98, except for “formal training” where the factor loading was 0.40. This item was subsequently removed as its removal improved the AVE of the factor from 0.30 to 0.40. The AVEs of the other factors ranged from 0.55 to 0.94. In terms of internal consistency, the composite reliability of all factors ranged from 0.69 to 0.97. In terms of the discriminant validity, there was no significant cross loading, the AVEs of all factors were higher than that of other factors according to Fornell and Lacker’s criterion [14] and the confidence interval for heterotrait-monotrait ratio (HTMT) for all items did not include the value of 1.0. The final structural model developed from PLS and its measurement results are detailed in Additional file 1: Figure S1. The forward translation version (after the reconciliation stage) as well as the backward translation version by an independent translator (after the harmonization stage) are tabulated in Additional file 2: Table S1. The final version of the validated questionnaire is shown in Table 2.

**Table 2:** The finalized version of the bilingual questionnaire to measure the perception and confidence towards cardiopulmonary resuscitation and automated external defibrillator training and placement strategy

Perception of AED placement strategies				
	Strongly disagree (1)	Disagree (2)	Agree (3)	Strongly agree (4)
The AED is clearly visible (*Peralatan AED jelas kelihatan*)				
The signage that shows the location of the AED is clear (*Papan tanda yang menunjukkan lokasi AED dipamerkan dengan jelas*)				
The AED is located in a location that is easily accessible at all times (including after office hours) *AED terletak di lokasi yang mudah diakses pada setiap masa* (*termasuk selepas waktu pejabat*)				
The steps in the AED instructional poster on how to use the AED are easy to follow (*Poster AED mempamerkan cara*-*cara menggunakan AED yang senang diikuti*)				
The AED is located at a secure site (*AED terletak di lokasi yang selamat*)				

Perception of importance of CPR and AED				
	Strongly disagree	Disagree	Agree	Strongly agree
CPR and AED are important in saving life (*CPR and AED penting untuk menyelamatkan nyawa*)				
It is important for an AED to be available in the place where I work (*Adalah penting supaya adanya AED di tempat kerja saya*)				
Using an AED is important on any unresponsive victims (*AED penting untuk digunakan ke atas mangsa yang tidak responsif*)				
AED practice drills should be performed on a regular basis (*Latihan mengendalikan AED harus dilakukan sebagai rutin tetap*)				

Concerns of injuring victims during CPR and AED				
	Strongly disagree	Disagree	Agree	Strongly agree
I am concerned of getting infection from the victim when performing CPR (*Saya risau akan dijangkiti penyakit daripada mangsa ketika melakukan CPR*)				
I am concerned of injuring the victim when performing CPR (*Saya risau jika tercederakan mangsa ketika melakukan CPR*)				
I am concerned of injuring myself when performing CPR (*Saya risau jika tercederakan diri sendiri ketika melakukan CPR*)				
I am concerned of injuring the victim if I use an AED device during CPR (*Saya risau jika tercederakan mangsa apabila saya menggunakan AED semasa CPR*)				
I am concerned in injuring myself if I use an AED device during CPR (*Saya risau jika tercederakan diri sendiri apabila saya menggunakan AED semasa CPR*)				

Concerns of legality in performing CPR and AED				
	Strongly disagree	Disagree	Agree	Strongly agree
I am concerned that I might be sued if I perform emergency CPR inappropriately (*Saya risau kemungkinan disabit kesalahan jika saya melakukan CPR secara tidak betul*)				
I am concerned that I might be sued if I used an AED inappropriately (*Saya risau kemungkinan disabit kesalahan jika saya menggunakan AED secara tidak betul*)				

Confidence and willingness to perform CPR and AED				
	Strongly disagree	Disagree	Agree	Strongly agree
I am confident to perform CPR (*Saya yakin untuk melakukan CPR*)				
I am confident to use an AED (*Saya yakin untuk mengendalikan AED*)				
I am confident in recognizing victim with no signs of life (*Saya yakin dapat mengenalpasti tanda*–*tanda tiada nyawa pada mangsa*)				
I will not hesitate to use an AED on an unresponsive victim (*Saya tidak teragak*-*agak untuk menggunakan AED ke atas mangsa yang tidak responsif*)				

### Discussion

The objective (1) perception of placement strategies of public access AED, was captured in Factor 1, labelled as “perception of AED placement strategies”; objective (2) perception on the importance of bystander CPR and public access AED is captured in Factor 2, labelled as “perception of importance of CPR and AED”; objective (3) perception on the confidence and willingness to apply these two lifesaving interventions is captured in Factor 5, labelled as “confidence and willingness to perform CPR and AED” and objective (4) the fears and concerns in applying these two interventions is captured in Factor 3 “concerns of infection and injuries during CPR and AED” and Factor 4 “concerns of legality in performing CPR and AED”.

To ensure a successful implementation of public access AED program, the preparedness of trained bystanders (with positive attitude and confidence) is as important as the placement of the AEDs itself [16, 17]. Whitney-Cashio et al. [10] suggested that AEDs should be placed in highly visible locations (“visibility”) that can be easily accessible (even after working hours) and with the “direction” and “signage” to access the AED should an emergency arises. Besides accessibility and visibility, AEDs should have clear instruction on how to use it and be placed in a secure area (e.g. with surveillance cameras) to minimize the risk of the AEDs being stolen (“security”). The corresponding item to measure each of these criterion in Factor 1 “perception of AED placement strategies” is listed in Additional file 3: Table S2.

## Limitations

The items in this questionnaire were mainly constructed and culled from published papers. In other words, there could have been other valid dimensions that also measures a specific factor or construct but are missed in our questionnaire. This is especially so in the construct of “concerns of injuring victims during CPR and AED”. In this construct, we merely measured the concerns or fears of injuring victims and contracting infectious diseases that may deter the initiation of bystander CPR and the use of AED. There may have been other fears and concerns that are valid and relevant but are not captured in this questionnaire.

## Supplementary information

**Additional file 1: Figure S1.** Path Diagram with values in the outer model representing factor loadings, values within the factors representing composite reliability and values in the inner model representing path coefficients.

**Additional file 2: Table S1.** Forward translation and backward translation of the validated questionnaire. This data describes the forward translation from the original English version to the finalized Malay language version (after the reconciliation stage where the two authors who translated it independently had discussed, resolved any discrepancy and reached a consensus) as well as the backward translation from the translated Malay language version back to the English version by another independent translator.

**Additional file 3: Table S2.** Criteria for AED placement strategy and the items that measure these criteria.

## Data Availability

The dataset used and analysed in this study are available from the corresponding author on reasonable request.

## Abbreviations

AED: automated external defibrillator
AVE: average variance extracted
CPR: cardiopulmonary resuscitation
EFA: exploratory factor analysis
CFA: confirmatory factor analysis
HTMT: heterotrait-monotrait ratio
ISPOR: International Society for Pharmacoeconomics and Outcomes Research
NMRR: National Medical Research Register
PLS: partial least square
SPSS: Statistical Package for the Social Sciences
UNIMAS: Universiti Malaysia Sarawak
