# Supplementary material for: Development, validation and translation of cardiopulmonary resuscitation and automated external defibrillator training and placement bilingual questionnaire
Source: BMC Res Notes. 2019 Oct 21;12:670. doi: 10.1186/s13104-019-4698-x (PMC6805342; doi:10.1186/s13104-019-4698-x)
Supplement: Supplementary file 1 — Additional file 1: Figure S1. Path Diagram with values in the outer model representing factor loadings, values within the factors representing composite reliability and values in the inner model representing path coefficients. [file 13104_2019_4698_MOESM1_ESM.docx]

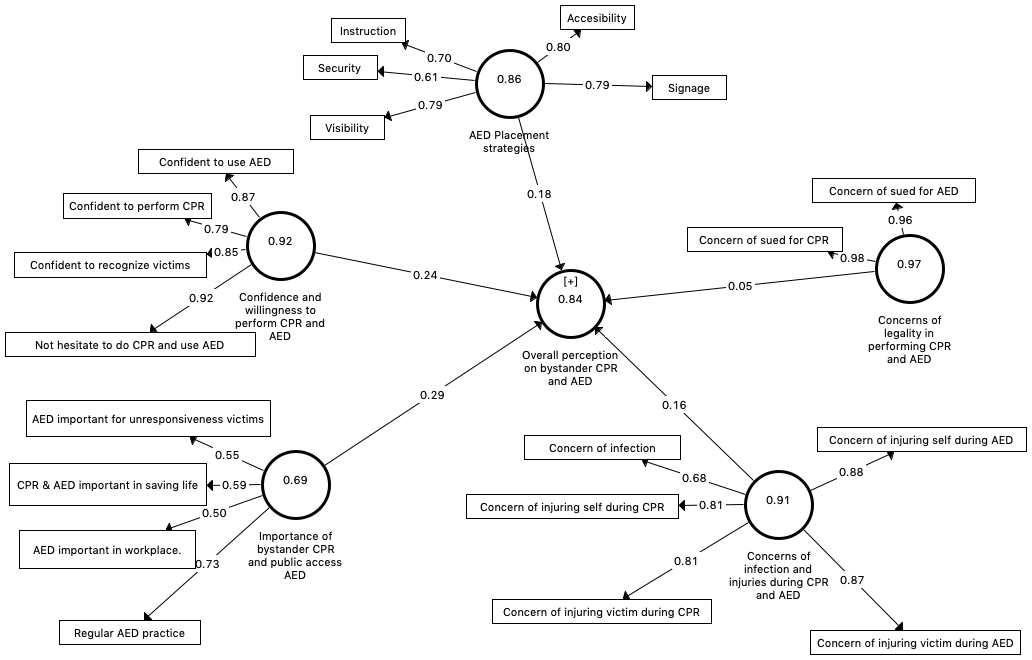


Figure S1. Path Diagram with values in the outer model representing factor loadings, values within the factors representing composite reliability and values in the inner model representing path coefficients.

Note:

1. The variance inflation factor (VIF) values for all factors are below 2.0, indicating no collinearity problem

2. Based on the path coefficients, “importance of bystander CPR and public access AED” is the most important predictor (path coefficient = 0.29, 95% confidence interval with bias corrected 0.07 – 0.311, p<0.0001), followed by “confidence and willingness to perform CPR and AED” (path coefficient = 0.24, 95% confidence interval with bias corrected 013 – 0.36). “Concerns of legality in performing CPR and AED” does not appear to be a significant predictor with path coefficient = 0.06, 95% confidence interval with bias corrected -0.05 – 0.17, p=0.24

3. The t-values for “AED placement strategies”, “confidence and willingness to perform CPR and AED”, “concerns of infection and injuries during CPR and AED”, “concerns of legality in performing CPR and AED”, “importance of bystander CPR and public access AED” are 2.47, 2.69, 0.71, 3.36 and 4.43 respectively. All values are above the critical values for significance levels of two-tailed 5%, i.e., 1.96 except for “concerns of legality in performing CPR and AED”.

4. R^2^ =0.27 indicating substantial predictive accuracy of the model

5. The effect size f^2^ values for “AED placement strategies”, “confidence and willingness to perform CPR and AED”, “concerns of infection and injuries during CPR and AED”, “concerns of legality in performing CPR and AED”, “importance of bystander CPR and public access AED” are 0.030.021, 0.002, 0.067 and 0.089 respectively indicating weak to medium effect size for all factors except for “concerns of legality in performing CPR and AED”.

6. The overall Stone and Geisser’s Q2 = 0.11 (above 0) indicating that there is predictive relevance of the factors in the model to measure perception
