# Supplementary material for: Development, validation and translation of cardiopulmonary resuscitation and automated external defibrillator training and placement bilingual questionnaire
Source: BMC Res Notes. 2019 Oct 21;12:670. doi: 10.1186/s13104-019-4698-x (PMC6805342; doi:10.1186/s13104-019-4698-x)
Supplement: Supplementary file 2 — Additional file 2: Table S1. Forward translation and backward translation of the validated questionnaire. This data describes the forward translation from the original English version to the finalized Malay language version (after the reconciliation stage where the two authors who translated it independently had discussed, resolved any discrepancy and reached a consensus) as well as the backward translation from the translated Malay language version back to the English version by another independent translator. [file 13104_2019_4698_MOESM2_ESM.docx]

Table S1. **Forward translation and backward translation of the validated questionnaire**

| **Construct** | **Original English version** | **Forward translation** | **Backward translation** |
| --- | --- | --- | --- |
| Perception of AED placement strategies | The AED is clearly visible. | Peralatan AED jelas kelihatan. | The AED device is clearly visible. |
|  | The signage that shows the location of the AED is clear. | Papan tanda yang menunjukkan lokasi AED dipamerkan dengan jelas. | The signboard that shows the AED location is clearly displayed |
|  | The AED is located in a location that is easily accessible at all times (including after office hours). | AED terletak di lokasi yang mudah diakses pada setiap masa (termasuk selepas waktu pejabat). | The AEDs are located in an easily accessible locations at all times (including after office hours). |
|  | The steps in the AED instructional poster on how to use the AED are easy to follow. | Poster AED mempamerkan cara-cara menggunakan AED yang senang diikuti. | The AED posters show easy-to-follow instructions on how to use the device. |
|  | The AED is located at a secure site. | AED terletak di lokasi yang selamat. | The AEDs are located in safe locations. |
| Perception of importance of CPR and AED | CPR & AED are important in saving life. | CPR & AED penting untuk menyelamatkan nyawa. | CPR & AED are crucial in saving lives. |
|  | It is important for an AED to be available in the place where I work. | Adalah penting supaya adanya AED di tempat kerja saya. | It is important that there is an AED in my workplace. |
|  | Using an AED is important on any unresponsive victims. | AED penting untuk digunakan ke atas mangsa yang tidak responsif. | AED is important for use on victims who are not responsive. |
|  | Person who handles an AED requires formal training. | Pengendali AED harus menjalani latihan formal. | AED handlers must undergo formal training. |
|  | AED practice drills should be performed on a regular basis. | Latihan mengendalikan AED harus dilakukan sebagai rutin tetap. | Training on the use of AED must be carried out on a regular basis. |
| Concerns of injuring victims during CPR and AED | I am concerned in getting infection from the victim when performing CPR. | Saya risau akan dijangkiti penyakit daripada mangsa ketika melakukan CPR | I worry about contracting diseases while performing CPR on victims. |
|  | I am concerned in injuring the victim when performing CPR. | Saya risau jika tercederakan mangsa ketika melakukan CPR | I worry about injuring the victim while performing CPR. |
|  | I am concerned in injuring myself when performing CPR. | Saya risau jika tercederakan diri sendiri ketika melakukan CPR | I worry about injuring myself while performing CPR. |
|  | I am concerned in injuring the victim if I use an AED device during CPR. | Saya risau jika tercederakan mangsa apabila saya menggunakan AED semasa CPR | I worry about injuring the victim while using the AED during CPR |
|  | I am concerned in injuring myself if I use an AED device during CPR | Saya risau jika tercederakan diri sendiri apabila saya menggunakan AED semasa CPR | I worry about injuring myself while using the AED when performing CPR on victims |
| Concerns of legality in performing CPR and AED | I am concerned that I might be sued if I perform emergency CPR inappropriately | Saya risau kemungkinan disabit kesalahan jika saya melakukan CPR secara tidak betul | I worry about the possibility of being convicted if I performed CPR incorrectly during an emergency |
|  | I am concerned that I might be sued if I used an AED inappropriately | Saya risau kemungkinan disabit kesalahan jika saya menggunakan AED secara tidak betul | I worry about the possibility of being convicted if I used the AED incorrectly during an emergency |
|  | I am confident to perform CPR | Saya yakin untuk melakukan CPR | I am confident in performing CPR |
|  | I am confident to use an AED | Saya yakin untuk mengendalikan AED | I am confident in using an AED |
|  | I am confident in recognizing victim with no signs of life | Saya yakin dapat mengenalpasti tanda-tanda tiada nyawa pada mangsa | I am confident in identifying signs of life in victims |
|  | I will not hesitate to use an AED on an unresponsive victim. | Saya tidak teragak-agak untuk menggunakan AED ke atas mangsa yang tidak responsif | I will not hesitate to use an AED on an unresponsive victim |
