# Supplementary material for: Development, validation and translation of cardiopulmonary resuscitation and automated external defibrillator training and placement bilingual questionnaire
Source: BMC Res Notes. 2019 Oct 21;12:670. doi: 10.1186/s13104-019-4698-x (PMC6805342; doi:10.1186/s13104-019-4698-x)
Supplement: Supplementary file 3 — Additional file 3: Table S2. Criteria for AED placement strategy and the items that measure these criteria. [file 13104_2019_4698_MOESM3_ESM.docx]

**Table S2 showing criteria for AED placement strategy and the items that measure these criteria**

| **Criteria** | **Items** |
| --- | --- |
| Visibility | The AED is placed in a clearly visible location |
| Signage | The signage that shows the location of the AED is clear. |
| Accessibility | The AED is located in a location that is easily accessible at all times (including after office hours). |
| Instruction | The steps in the AED instructional poster on how to use the AED are easy to follow. |
| Security | The AED is located at a secure area. |
